# Stakeholder Perspectives and Values when Setting Waterbird Population Targets: Implications for Flyway Management Planning in a European Context

**DOI:** 10.1371/journal.pone.0081836

**Published:** 2013-11-26

**Authors:** James H. Williams, Jesper Madsen

**Affiliations:** 1 Arctic Research Centre, Department of Bioscience, Aarhus University, Aarhus, Denmark; 2 Communications & Systems Department, The Open University, Nottingham, United Kingdom; University of Kent, United Kingdom

## Abstract

Managing and controlling wildlife species within Europe is an acknowledged part of conservation management, yet deciding and setting a population target in order to control a population is perceived to be conceptually very challenging. We interviewed stakeholders, within a variety of governmental and non-governmental organizations, to evaluate their perspectives about setting population targets as part of waterbird management for controlling population sizes. We conclude that the setting of a quantifiable population target is beneficial as a measurable objective for monitoring and evaluating management actions. However, it must be recognised as just one possible measurable objective and there may well be multiple supporting objectives that encapsulate the management aims of different stakeholders. When considering wide-scale control of waterbirds species, where it is likely that population size matters, any population target should be coupled to the issues being addressed. We highlight that it is important to actively engage with stakeholders as part of the decision-making process, not only to gain consensus but to share knowledge. A clear understanding of the context and the rationale for controlling a waterbird species is needed to align the interests of diverse stakeholders. The provision of scientific data and the continuous monitoring of management actions is viewed as beneficial and demanded by stakeholders, as part of any decision-making process when setting population targets. This facilitates effective evaluation of management actions, helping managers make wise decisions as well as enabling the continued development of management plans.

## Introduction

For several decades the focus for wild bird conservation and management within Europe has been to safeguard species and habitats, to ensure the long-term preservation of Europe's biodiversity and its bird populations. One of the cornerstones of EU conservation policy has been ‘The Birds Directive’ which affords protection to all bird species naturally occurring within the EU [1]. Many bird species have benefitted and, in particular, migratory geese have prospered with most populations having increased dramatically over the past few decades [2]. The success of geese has increasingly focused attention on the environmental and social-economic implications of burgeoning goose populations which have progressively come into conflict with farmers, who sustain crop damage [3], [4]. The African-Eurasian Waterbird Agreement (AEWA) now promotes the development of management plans for waterbird species that cause significant damage [5]. In the case of the Svalbard pink-footed goose *Anser brachyrhynchus* there have been calls to control the size of the entire population due to an escalation of agricultural conflicts during its spring migration through Norway. This has led to the development of the first International Species Management Plan (ISMP) within Europe, under the auspices of AEWA. Based on the principals of adaptive management it includes the setting of a sustainable population target for the Svalbard pink-footed goose [6]. Its goal is to maintain the population in a favourable conservation status, whilst taking into account economic and recreational interests. It was agreed to maintain the population at a target of around 60,000 individuals, employing hunting as a management tool to stabilise the population, in order to realize a series of ISMP objectives including the alleviation of crop damage and arctic tundra degradation [6].

Setting population targets is often done in the sphere of biodiversity conservation. Current theory and practice predominantly focuses on determining minimum population targets to prevent extinction or set targets for recovering species. The setting of population targets for wildlife species is viewed as problematic and somewhat contested (e.g. [7]–[10]). Some conservationists have stated that science alone must drive the process for setting measurable management conservation objectives and should be insulated from value driven processes [7]. Other conservationists recognize that social and economic factors should be accounted for and consider subjective values and politics an inherent part of setting conservation targets [10]. This dilemma has implications when considering the adaptive management of waterbird populations. Setting wide-scale population targets for controlling waterbird species, such as pink-footed geese, are rare within Europe. Adaptive harvest management has been applied widely and systematically in North America for the last two decades to maintain or control waterbird populations [11]. However, policy makers have refrained from its application for waterbird management within Europe. It has been argued that variation in national policies, cultural and societal viewpoints within Europe could hinder the development of such a management system as agreement on objectives and management actions, for example, would be impossible to attain [11].

Adaptive management provides a framework for decision making which is suited to situations where there are potential sources of difficulty in making management decisions, in particular: (1) disagreement about appropriate management objectives and (2) uncertainty about an ecological system and the impact of management actions [11]. It requires a formal and structured process to reduce these uncertainties through iterative monitoring and learning that improves management over time [11]. Adaptive management promotes the participation of different stakeholders to agree goals and management objectives. It encourages stakeholder groups to learn from each other helping management policy to reflect a range of different stakeholder values and viewpoints [12], [13], [14]. Accordingly, it is suggested that multi-stakeholder participation in adaptive management is an effective way of capturing the information and perspectives necessary to manage social-environmental systems. This can lead to better management plans and has the potential to make environmental management more democratic [14]. Furthermore, adaptive management calls for targets to measure the success of management actions, linking actions to target conditions as part of ‘cause-and-effect chains’ [12]. It has also been stated there is a need to differentiate between goals and objectives in conservation management. While goals are broad and visionary, management objectives should be measurable and explicit to effectively evaluate progress of management actions and enable stakeholders to make wise choices and justify these [7], [8]. Nevertheless, establishing biodiversity targets and the question of “How much is enough?” has been vexing wildlife managers for several decades; providing numerous challenges in establishing explicit quantifiable targets, in applying science to policy and translating policy into action. In addition, a single and absolute answer has been regarded as detrimental with targets needing to be communicated as hypotheses [7].

The involvement of a variety of stakeholders and the need for agreement on management objectives raises questions about the influence of societal values and different perspectives in the decision-making process when setting measurable objectives, such as a population target. It has been noted that the setting of conservation targets given social, economic and political influences is an unsatisfactory compromise with pragmatic concerns on feasibility over-riding biological risk assessment [8], [9]. A multitude of factors are likely to help or hinder gaining agreement on a population target due to stakeholder diversity and their differing perspectives, values and goals. Although it is generally accepted that collaborative and participatory governance leads to more sustainable and effective environmental policy, it has been stated that it is the preferences of ‘actors’ actually involved in the decision-making process which predominately determine outputs and outcomes [15].

Differing stakeholder perspectives about setting population targets for waterbirds and how they interact are not fully understood, as there is limited data available in published literature. This study set out to investigate and understand the diversity of values and viewpoints amongst a range of international policy and decision makers about setting population targets for waterbird species, particularly geese. By assessing different stakeholder perspectives the intention was to identify possible convergent or divergent perspectives, which would help or hinder the process of collaborative management and establishing a population target as a measurable management objective. Central within an adaptive management decision-making process is the need for agreement on management objectives, which prompted the following research question: What are stakeholder perspectives on setting population targets for waterbirds, and how might these influence gaining agreement on a population target within an adaptive management framework? This was further explored by formulating the following questions: (1) What is the management goal and how is this influenced by stakeholder perspectives? (2) Why set a population target, what does a population target represent and is one useful? (3) Why manage a population and what justifies lethal control? (4) Who and how should a population target be decided?

The authors are part of an international working group who developed the ISMP for the Svalbard pink-footed goose. Investigating perspectives on setting waterbird population targets was of considerable interest to those involved in this process, as the setting of a population target for controlling a species was a much debated issue. This study was instigated out of a desire to explore current academic thinking and gain insights into stakeholder perspectives, so that this learning could help guide those involved in similar decision-making processes in the future. The viewpoints expressed in this paper do not necessarily represent the collective or official view of the group.

## Methods

### Study design

In order to answer the posed research questions a qualitative research study was considered the best means to do this. Qualitative interviews enabled more comprehensive data to be obtained which permitted detailed analysis for a better understanding of stakeholder perspectives. This approach provided the data needed for a broad explanation of the issues whilst identifying key themes that highlighted differing stakeholder perspectives and how these might influence decisions on management objectives and actions.

### Ethics

We sought advice regarding ethical approval from the appropriate committee (The Open University Human Research Ethics Committee) and the research protocol for the study was submitted for ethics review and approved. Formal written approval was obtained from the ethics committee Chair (ref: HREC/2013/1369/Williams/1). Consent to participate was voluntary and was obtained by email. Participants were advised of the nature of the study and given written details of questions to be asked prior to interviews, along with the involvement of the authors in the ISMP for the Svalbard pink-footed goose. All participants gave written informed consent to take part in the study. Anonymity and confidentiality of the interviews were guaranteed to all participants.

### Interviews and sampling

A series of semi-structured telephone interviews were undertaken amongst a selected group of international policy and decision makers. These were individuals with specialist knowledge and involved in waterbird management within a variety of governmental and non-governmental organizations. The structure of these interviews was based on a series of questions exploring the topics below.

### 

Interview topics included:

- Perspectives and values associated with the conservation and control of waterbird species?
- The function and role of population targets?
- What factors/data/scientific information should be considered when determining a population target e.g. biological, social and economic factors?
- Perceptions of the use, dissemination practices, relevance and quality of scientific information and population modelling?
- Perspectives on the decision-making process to set population targets. Who should be involved and how to engage a range of stakeholders?
- Perceptions of the roles, influence and possibility of conflicts between scientists, policy makers and other actors involved in the decision-making process when setting population targets?

Purposive sampling [16] was used to select participants according to the following criteria to give a matrix sample structure:

3 geographical regions

- EU/Range states of the Svalbard pink-footed goose (Norway, Denmark, The Netherlands and Belgium)
- Scotland/UK (where strategic goose management is practised)
- US (Greater Snow Goose flyway and where target-setting is applied)

4 representative groups

- Nature/environmental agencies
- Conservation representatives
- Hunting representatives
- Farming representatives

At least one organization was targeted to be contacted for each the four representative groups, within the selected European countries (the main focus of this study). Interviews with North American governmental agencies were primarily sought to gain insights from their experiences when setting waterbird population targets. Decision and policy makers were identified by the authors using publically available data or by referrals from the authors' contacts. Not all categories could be assigned a prospective respondent, as either a representative organization did not exist, contact details or an appropriate person were unavailable. Recruitment for the interviews was conducted by the authors by email and telephone. None of the respondents contacted declined to be interviewed.

A total of 26 semi-structured telephone interviews were undertaken (Table 1), representing a broad range of those involved in waterbird management within Europe. Further interviews, with a limited number of possible organizations and personnel, were considered unlikely to provide further insights. Interviews were prearranged and carried out between the 31^st^ January and 23^rd^ February 2012. They lasted approximately 40–45 minutes and were recorded. Interviews were transcribed and then manually coded using Microsoft Word and Excel. Codes were phrased as closely as possible to the original data, developing key words for coding interview transcripts. These codes were iteratively reviewed and agreed by the authors for consistency and were used to manually sort quotes and phrases into themes from which the results were derived, accordant with double-coding and grounded theory practices [17] [18]. The responses from individuals within the four stakeholder groups have been used to illustrate, in part, their collective ‘frames of reference’ [19]. These incorporate some of the beliefs, assumptions, norms, values and issues that emerged amongst interviewees based on their experiences. They represent the apparent viewpoints that influence their understanding of the situation, their response to it and preferred solutions. The results of this study are drawn from the qualitative interviews undertaken but published material was also accessed to confirm statements by some interviewees and provide background information (see references).

**Table 1:** Number of respondents interviewed within planned sample structure. The lower case letters in brackets have been used as codes to identify interviewee quotes.

Region	Nature/environmental agencies (w)	Conservation representative (x)	Hunting representative (y)	Farming representative (z)	Total
EU (a)	1	1	1	-	**3**
Norway (b)	1	-	-	1	**2**
Denmark (c)	1	1	1	2	**5**
The Netherlands (d)	-	1	1	2	**4**
Belgium (e)	1	1	-	1	**3**
Scotland/England (f)	2	2	1	-	**5**
Canada (g)	2	-	-	-	**2**
US (h)	2	-	-	-	**2**
**Total**	**10**	**6**	**4**	**6**	**26**

## Results

### What is the management goal and how is this influenced by stakeholder perspectives?

There was general consensus amongst interviewee stakeholder groups on the overarching goals for wildlife management and these were also applicable to waterbirds. However, there were subtle differences in their frames of reference and subsequent management objectives and desired outcomes, particularly, when managing species where population sizes were causing management issues. The following are vignettes based on interviewee responses:

Government agencies: For representatives of government agencies the goal for wildlife management was to maintain biodiversity and the ecological integrity of natural habitats. Their focus was on conservation and the protection of natural habitats and wildlife species. This was underpinned by obligations to fulfil both national and international legislative requirements. Their responsibility was towards ensuring biodiversity but they recognised societal and economic interests. Where these conflicted with their objectives statutory principles governed management responses.

*“Our goal is to have healthy eco-systems” (bw interview); “My agency administers the hunting and wildlife legislation and nature protection laws, for instance working with wildlife reserves, which are a major part of what we do to protect species. Part of what we do to meet the obligations in the EU directives” (cw interview); “From the outset the Birds Directive sets a framework for all species and its primary objective is conservation not population regulation. There are all kinds of sensitivities in relation to population control. Our focus is where there is damage there is possible justification. You have to exercise proportionate control measures that will actually deal with the problem. The legislation accepts we may need to derogate from protection requirements in relation to agriculture and fisheries conflicts and air hazards etc.” (aw interview)*

Conservationists: Similarly, for representatives of conservation organizations their goal and focus for wildlife management was preservation, maintenance and protection - ’conservation‘, to ensure the continued existence of species within healthy ecosystems. As one interviewee stated *“waterbirds are part of biodiversity and there is a long tradition within conservation management to maintain this [biodiversity]” (ex interview).* Their perspectives often intertwined appreciating nature for its inherent worth, aesthetic values and ’instrumental’ benefits that wildlife and birds convey to their members and society. Flourishing wildlife and increasing populations of waterbirds, such as geese, were for conservationists a very positive sign; a success of conservation and protection.

*“They [geese] have their own right to live, their intrinsic value and we, humans, must respect nature for that” (ex interview); “If you provide good habitats then you get the birds and other wildlife. Geese have huge recreational meaning for our members and the public.” (dx interview); “Nature should be protected for its own sake and it's important that the public can experience wild nature. Species should be offered enough space for resting and feeding and society needs to provide this space” (cx interview)*

Farmers: Farming representatives readily stated that ensuring biodiversity was an important goal and recognised the value of healthy ecosystems, not only for wildlife but for farming as well. They also stressed there was a need to account for the impact of certain waterbird species, in particular rapidly growing goose populations. Underlying this view were economic concerns about protecting the productivity of their farmland, maintaining farming livelihoods and even the integrity of viable rural communities. For them the goal for wildlife management involved striking a balance between economic interests and society's desire for biodiversity in a human managed environment. Nevertheless, some farmers indicated that if society was willing to pay compensation this would mitigate any economic losses due to crop damage.

*“Farmers depend on healthy nature” (bz interview); “It's very important to preserve all kinds of species, don't have a problem with that at all. It's also in our interest to have good nature” (ez interview); “When populations [geese] have been growing as much as we have seen in the last 10–15 years, then this is a problem for farmers economically, there's the cost of scaring and loss of crops. For some farmers this is very costly they might have to give up farming” (interview cz); “It's very difficult for farmers to take care of the situation and safeguard his land. It is so overwhelming when 1000 birds just come one day on a field, stay for a week and then leave. What is left is that production is reduced by say 25%” (bz interview); “If society compensates and helps to accommodate the geese for the number they want, I don't think there would be any problem” (cz interview).*

Hunters: The focus for wildlife management amongst hunting representatives was to maintain habitats and species, primarily for the benefit of their activities. They valued nature and biodiversity but it was the utility they derived from nature which drove their perspective, hunting for them is a recreational pastime. These representatives believed that hunters have a role to play in wildlife management and had a strong desire for this to be recognised. They also accepted they must bear the responsibility for their actions. Furthermore, they considered there was a long tradition of hunters' managing wildlife species for their sustainable use, which they linked to a conservation ethic. From their perspective conservation should not be viewed as synonymous with rigid protection.

*“Naturally we like to go hunting and we like to shoot wildlife species but we have a passion for wildlife and it is very important for us that different populations are in good health. We only want to hunt species that are in a sustainable state.” (cy interview); “We have an international not just a national responsibility for nature conservation and biodiversity and to society of course. I think we are doing that as hunters. It's a combination of sustainable hunting and shooting for reduction of crop damage or, for example, the safety at airports.” (dy interview); “Hunting needs to fit to the reality of the landscape, how we manage landscapes and the new ways society approaches it. Managing for long-term sustainable yield is a much better way of approaching it from our perspective. One of the problems in Europe is that protection instead being seen as the means, it is seen as the end. People have mistaken protection as conservation and it is only a part of conservation.” (ay interview).*

Ensuring biodiversity and healthy eco-systems were certainly accepted goals for wildlife and waterbird management, amongst these interviewees. However, these vignettes illustrate that stakeholders often looked at the context of situations to be managed in a variety of ways, considering different aspects more or less important depending on the outcomes and issues that were of concern to them. This has implications for aligning goals to management objectives where different stakeholders perceive there to be different priorities for management objectives and actions, as well as alternative ways to achieve their overall goals.

### Why set a population target, what does a population target represent and is one useful?

For those involved in conservation and wildlife management the need for setting population targets was generally accepted as beneficial and as a means to monitor the success or failure of management actions.

Government agencies: *“It's good to have reference values for species so we can understand whether they are in a favourable conservation status” (aw interview)*

Conservationists: *“Yes targets are really important. Without knowing what you are trying to achieve, then how can you focus on it” (fx interview); “Objectives should be measurable so we can look, 5 years later on and say how did the population react and what parts of the management plan did the population react upon. Measuring and monitoring is important.” (cx interview)*

These comments also highlight how a quantifiable objective, such as a population target, is then associated with the management intent and the desired outcomes of those involved in the decision-making process. For instance, when talking about ensuring biodiversity and promoting the recovery of particular bird species government agency and conservation representatives were comfortable with setting minimum population targets. However, they were cautious and very reticent about setting maximum population targets. Whereas, some farming representatives considered a maximum population size as an indicator and focus for the issues they faced, specifically in relation to crop damage. Nevertheless, responses to setting a maximum population target were more nuanced with concerns expressed about the reality of trying to realise a maximum population size.

Government agencies: *“We have agreed minimum populations, for species such the Greenland white-fronted goose but it is a different conceptual level when setting a target that population shouldn't go above. We have rather shied away from setting national targets [upper]. It's better to focus on what really is the problem we are trying to fix, rather than hung-up on a number” (fw interview).*

Conservationists: *“We are nervous about setting an upper limit. Is it our role to set upper limits for species so long as we do not take the problem seriously, by that I mean providing space to enable geese and farmers to coexist.” (cx interview); “As a conservation organisation, we do not consider it appropriate to set maximum population sizes for protected species. This is too simplistic a measure. It is far more appropriate to measure the damage allegedly being caused.” (fx interview)*

Farmers: *“Maybe a population of barnacle geese of say 300,000 would be a good start [to manage issues of crop damage], of course monitoring to see how they cope with that size. Humans are controlling everything anyway so why not have a top number on a goose population” (cz interview); “We are in favour of setting negative targets, let's say no more than this number. We also are realistic, we see no way to realise this now numbers are so massively grown. I believe there is no way to control it [population] by setting a maximum number, maybe we can control the growth” (dz interview).*

The setting of a maximum population target is contentious but what underlies this as an area for debate is conceptually linking it to the management issues to be addressed and as a desired outcome. Attributing a single numerical value to a complex situation is laden with issues, as one interviewee stated it is *“more complicated than a single number” (fx interview).*

### Why manage a population and what justifies lethal control?

A core issue that emerges when setting a population target for a waterbird species is establishing it as a unifying objective where there may be a variety of underlying management objectives and issues. Alternative frames of reference can lead to different objectives, concerns and possible ways to manage issues. When considering population control these varying perspectives can generate debate about: what are the issues, does controlling a population and setting a quantifiable population target resolve these? It is at this point that perspectives started to diverge, as to how issues such as agricultural damage can or should be tackled.

The farming representatives interviewed generally considered that crop damage due to increasing goose populations could be alleviated, to some extent, by population control. For them it was the scale of the problem that daunted them, whilst alternative management actions were regarded as ineffectual. For them lethal population control was seen as a viable solution to the issues they faced.

Farmers: *“There are 3 or 4 species, mainly geese, which have grown in population so fast and are so enormous in numbers now we fear there is no end to it. The damage to agricultural production is gigantic and since a few years ago there is a lot of damage to nature areas too. Everybody knows something has to happen. We are trying all different kinds of methods to manage the population. You can treat the eggs, shoot them etc. You have to use a method to manage the population and, in my view, it is to kill and eat them” (dz interview); “Scaring costs a lot in time and effort and then you scare them to other parts and make more damage there. Hunting is another way to do it. It both scares them away and you get something out of it e.g. for hunters it's nice to shoot some geese and get something for the pot” (cz interview)*

Those representing conservation organizations tended to question the premise that addressing issues of agricultural damage could be done through population control. As one conservationist interviewee stated *“we shouldn't control populations we should control the habitat” (dx interview).* This is an indication of an alternative frame of reference, whereby the population of a species is not the issue but the carrying capacity of the habitats that support it. It was accepted that human intervention was needed in certain problem situations but there was a strong preference for intervention to address issues locally and the use of lethal control considered a last resort.

Conservationists: *“When you have very nutrient poor ecosystems and you have a lot of geese damaging the eco-system we can imagine some sort of population control. On the other hand there are situations where there are social problems e.g. safety related to airplanes and traffic. In these situations we can also agree on regulating a population but only at a very local and focused scale to get rid of the problem and not the birds.” (dx interview); “The need to control must be very strong, first look to all other possibilities to solve the problem with the option to control a population a very last resort”. (ex interview); “We wouldn't consider it appropriate to manage a population unless there were proven serious conflicts with other interests. In essence our approach is predicated on the legal requirements set out in the Birds Directive.” (fx interview)*.

For government agencies legal obligations underpin their conservation objectives and management actions. For example, the EU ‘Birds Directive’ establishes a comprehensive scheme of protection for all wild bird species, banning their deliberate killing and capture [1]. For government agencies within the EU, as well as conservation organizations, this is a fundamental principle of conservation management. As a government agency representative stated the *“Birds Directive sets a framework for all [bird] species and its primary objective is conservation not population regulation” (aw interview).* The Birds Directive does recognise the legitimacy of hunting but sets out a scheme of provisions to regulate it and lists specific ‘huntable’ bird species. Furthermore, the directive allows for exceptions (derogations) permitting lethal control of any bird species under strictly specified conditions. Nevertheless, cautious responses were given by government agency representatives in relation to managing certain species where population sizes were potentially an issue. The rational and actions needed for controlling a population were very dependent on the underlying causes, issues and the scale of the problem. For instance population control of an often cited ‘conflict’ species, the cormorant, was countenanced under EU directive derogations but this was on a limited scale [20].

Government agencies: *“Yes we mainly control to lessen the conflict with fishing interests but also towards other species, e.g. salmon in Ringkøbing Fjord. We don't have a population target for the cormorant; it's more that we try to control the conflicts where they are. That of course effects the overall population but that is not the goal.” (cw interview); “The degree of control depends on species and what kind of damages and what their population trend is. Can't look just at population size have to look at actual damages and actual conflicts” (bw interview)*.

All interviewees accepted these legislative principles, which govern the types of situations when lethal control was justifiable. Where viewpoints started to diverge was attributing the cause of a problem situation to the size of a population and the use of lethal control to manage these situations. These legislative principles do recognise that adverse biological, social and economic impacts of a bird species should be accounted for. The question is: when and at what scale do these factors become an issue, requiring a population to be controlled? Amongst government agency representatives there was recognition that the size of certain waterbird species was becoming an issue but there was a preference for managing issues locally and the use of lethal control needing careful consideration. Concerns stemmed not only from ecosystem and management uncertainties but also from political considerations. A number of interviewees expressed this in a variety of ways, for instance concern about the setting of precedents (primarily for maximum targets), the difficulty of communicating targets to the public and the consequences of failing to achieve them.

Government agencies: *“There comes a time, e.g. North American white goose, where we have to think about population level control for serious impacts on natural systems or conflicts with farmers. We may have to actively reduce the population as a whole. In the UK we have not reached that situation. It would simulate a lively conversation with a lot of stakeholders were we to head in that direction. Ironically the closest is the Canada goose but a non-native does not pose quite the same moral or conservation issues as wide-scale population control, or to drive numbers down locally” (fw interview); “At least today's population [pink-footed goose] is seen as large and potentially having a negative impact on the arctic environment, we are starting to see the impact of grubbing. If you have an action plan to control a species it's a good idea to have population target to scale the effects or not, so you can see if hunting is having a large effect and can then adjust measures.” (bw interview); “I think it will be necessary [population control] for some species in the future. We are a bit reluctant to do this because we need to have some insurance that we are actually able to control a population and the problem when we set targets, otherwise we will have a lot of criticism”. (cw interview)*

This analysis highlights that, in relation to population control, the scale of issues and potential management actions to remedy these do generate underlying tensions related to responsibility as well as ‘controllability’. Looking at this from an ethical perspective this has two potential dimensions: 1) Capacity to act/entitlement 2) Accountability of actions/duty of care.

The first point is best illustrated by a response from a conservationist interviewee: *“Ethically in our organization we feel we can't put a top level on a population, how can we say 100,000 are too many for example as a top limit for a population” (ex interview)*. This statement is underpinned by intrinsic values, questioning the entitlement to intervene. However, managing problem situations by lethal control was accepted by some conservationists as an option, so long as legislative principles were followed: *“We would only consider it appropriate to manage a population of a native wild bird species if it was for one of the above reasons i.e. within the legal framework of the Birds Directive.” (fx interview)*.

The second is linked to the ability to regulate hunting, ensuring it is sustainable. There was a degree of concern expressed by some conservationists, in part, based on historical experiences. As a conservationist interviewee commented *“past declines are a result of the destruction of habitats but also of over exploitation” (ax interview).* Such views then translate in to a desire to closely monitor any efforts to control a wildlife population by hunting, viewed as plausible at a local scale. In addition, when talking about population control there was uneasiness about publically expressing large numbers, particularly for government agencies and conservation organizations concerned about public reactions and unintended consequences:

Government agencies: *“At a local level you can liaise with land managers and hunters and get an idea of what is happening working with people on the ground, so some kind of adaptive management at local level is possible but it won't work if you scale up to a national level” (fw interview);. “How you deal with this in the context of going public is difficult. Do we say we are going to reduce our cormorant population by …. I am very cautious once you go down this route bringing populations down by the order of 20–30%, we open up bigger vista by way of other people saying we can start on other populations”. (aw interview)*

Conservationist: *“Many people [the public] would not agree to 50% of the population being shot” (dx interview).*

For further insights it was useful to draw on North American experiences as a ‘frame of reference’ as to when wide-scale control of a population has been considered necessary and acceptable. The stated goal of the Greater Snow Goose Flyway Management Plan: *“is to sustain the greater snow goose population at a level that maximizes a balance between benefits to society and habitat integrity”* [21]. From interviewing US and Canadian wildlife managers, involved in the management plan, the need for action was driven by concerns for the arctic environment and its degradation. The scientific evidence gathered had demonstrated the adverse impacts of rapidly expanding goose populations on this fragile environment. The scale and consequences of this problem were considered significant enough for intervention at a population scale. Also of importance were the objectives to minimize crop damage and maximize other human-related benefits, such as hunting and wildlife viewing [21], [22]. It is apparent, from interviewees and management publications, that the acceptability of this plan and its stated population targets (a target range) was founded predominantly on ecological concerns and backed by science but with clear recognition of stakeholder interests. Sustained engagement, explanation and dialog with stakeholders and the public was seen as vital in gaining this general acceptance [21], [22].

This North American example and the perspectives of the stakeholders interviewed here illustrate that the ‘issue of scale’ spans spatial, temporal and numerical boundaries of acceptance. The inference is that the reasons for intervention, to manage a population, need to be justifiable but in addition management actions must be proportionate and acceptable to the majority of stakeholders and the public. The perspectives of different stakeholders can influence how a population target is perceived to be linked, as measurable objective, to a desired goal when managing a particular set of issues along with what are considered as appropriate actions. The role of stakeholders in the decision-making process raises questions about whom and how a population target is established and realised. As one conservationist interviewee stated: *“There are narrow situations where population targets are appropriate. The key to success would, in my view, would be clear setting of targets and clear communication of these and the reasons behind them. Stakeholders should then be consulted on how best to reach these stated goals.” (fx interview)*

### Who and how should a population target be decided?

When questioned about who was ultimately responsible for setting population targets there was general agreement amongst non-governmental interviewees that it was the role of the relevant government authorities to lead the decision-making process; predominately at national levels but also internationally where appropriate. Non-governmental interviewees did not want the authorities to simply prescribe targets and solutions. Rather they were there to facilitate the process by providing resources (expertise, information and finance etc.) and to establish policies for engagement and action when responding to problem situations. There was a strong desire by interviewees to be involved and for stakeholders to have their say when authorities set policies.

Conservationists: *“National governments; they should be held accountable for doing so through legal instruments but all stakeholders: conservationists, wildfowlers, landowners, government agencies etc. should be involved.” (fx interview)*

Farmers: *“Easy to point to the government of course. Governments are effectively responsible for policies and they should be the organization to set a target but only, of course, after elaborate discussions with all stakeholders.” (dz interview); “It's the government organizations who should decide. They should be neutral as they have the knowledge of populations and issues, of course in cooperation with locals and others [stakeholders] but they [governments] should be the main source of knowledge and support for the process.” (cz interview)*

Hunters: *“It should be led by inter-governmental agencies in consultation with key stakeholders. It should be a consortium; things have to be talked through. Start with science and then go into the other criteria. Can't only be responsible to science there are political decisions to be made and compromises depending on different interests.” (ay interview)*

All interviewees agreed that when setting population targets the decision-making process should involve multiple stakeholders. In addition, interviewees regularly referred to the role of science in helping to determine population targets and the need for monitoring to evaluate the impact of management actions. The rational for management actions must be based on factual knowledge underpinned by scientific expertise as acknowledged and demanded by interviewees, although some expected local knowledge and skills to be recognised and valued as well.

Government agencies: *“Need to have good scientific base and have good scientific information and need to communicate that and the arguments, if you want to be heard. You need scientific facts from all areas e.g. biological, agricultural and society.” (bw interview)*

Conservationists: *“You needed monitoring, it is fundamental. It should be reliable, regular and representative for entire population. If not it is risky and difficult to set targets and agree on them.” (ax interview)*

Farmers: *“If you have a target you also have to monitor it, for instance monitoring is important to see how much damage they do” (dz interview)*.

Hunters: *“Yes you can set targets but this needs research, we have to have data on the population and breeding success etc. Important factors are the breeding success and the growth of the population but also the damage they [geese] cause to traffic, to nature and other species; these are important indicators to monitor as well.” (dy interview)*

Some interviewees suggested the actual setting of targets should be done by conservation scientist alone but as one government agency interviewee commented *“it's not just the scientific community other stakeholders have views that need to be taken on-board” (aw interview).* As indicated by a conservationist interviewee the role of science is precursory and the setting of a population target is not exclusively a scientific decision; it is a social and political decision as well: “*Scientific and information driven methods helps us to decide a desired population status but this scientific method should serve the purpose of a long term political vision*” *(ax interview)*. From these interviews the inference is that science provides the basis for setting population targets, providing facts and evidence to support the rational for decisions. It is then for stakeholders to make their judgements collectively and then governments to establish the relevant policy and management frameworks to achieve desired goals, objectives and outcomes.

## Discussion

This study was focused on the research question: What are stakeholder perspectives on setting population targets for waterbirds, and how might these influence gaining agreement on a population target within an adaptive management framework? Reflecting on this, using the four supportive questions has provided a number of useful insights. Firstly, ensuring biodiversity and healthy eco-systems was widely accepted amongst interviewees as a goal for wildlife management. Nevertheless, when discussing issues related to burgeoning populations of some waterbird species what emerged were differing stakeholder perspectives on management objectives, priorities and how to respond to issues, such as crop damage. These differing ‘frames of reference’ consequently guided interviewee responses to subsequent questions.

In response to questioning about the usefulness and purpose of setting population targets interviewees generally recognised them as being useful as: quantifiable objectives, helping to set priorities, assess the status of a population and as a farmer representative stated “*in terms of public relations setting a target is a good idea because it focuses the mind*” *(dz interview)*. Goal-orientated behaviour associated with targets is not necessarily rejected or contested. The difficulty for many interviewees, especially amongst government agencies and conservationists, was conceptually linking a species population size as a unifying target to a variety of underlying issues with associated management objectives and actions, as reflected in the statement; “*it's more complicated than a single number*” *(fx interview)*. This is not to say that setting a population target is not relevant or useful but the inference from interviewee responses was a desire to link measurable objectives to specific issues. Does a population target hinder agreement amongst stakeholders by masking differing objectives? Or can it represent a consensus point for broad and visionary goal as well as a measurable objective [7]? The indications from this study are that, as part of an adaptive management process, a population target can be conceived as a quantifiable expression for a stakeholder endorsed management objective, for a given set of circumstances. If supported by a hierarchy of multiple objectives it can help stakeholders to evaluate and decided upon appropriate actions to realise a variety of objectives that still achieve an agreed/stated overarching goal. Nevertheless, differing stakeholder goals, objectives and perspectives do have implications for setting and gaining agreement on a quantifiable population target.

When setting population targets interviewees in this study believed that it was the responsibility of government authorities, at national and international levels, to lead the decision-making process and set policies to achieve agreed objectives. Interviewees expected government authorities to develop policies and make decisions based on wide-ranging stakeholder involvement and collaboration. What is problematic, in relation to controlling waterbird species, is gaining consensus where legal obligations, moral values, economic interests and the scale of perceived issues clearly shape stakeholder perspectives. These in turn can lead to divergent viewpoints as to: when and why intervene and what are appropriate, desirable and proportionate management actions to manage particular problem situations. This is an issue for government agencies endeavouring to align the interests of multiple stakeholders in order to define, agree and achieve waterbird management policies, goals and objectives, especially where there are differing perspectives.

Within Europe the “participation of stakeholders in environmental decision-making” is one of the Three Pillars outlined in the Aarhus Convention to enhance environmental governance [23]. Furthermore, stakeholder participation as part of adaptive management is regarded as a prerequisite for developing management policies that reflect a range of different values and viewpoints [14]. However, it has been noted that the interests and political goals of those ‘actors’ actually involved in any participatory decision-making appear to be the most important causal factor in explaining policy outputs [15]. In addition collaborative agreements often lead to a compromise between the competing interest of ‘actors’, rather than seeking ecologically optimal solutions [15].

These issues raise a number of important points for consideration when setting population targets, especially with regard to population control. Those involved in setting a population target for controlling a species should regard it as a social construct. A population target can be conceived as a unifying measurable objective, intended to reflect a variety of stakeholder objectives, but those involved in deciding must recognise that these objectives are influenced by legal obligations, ecological imperatives, human values as well as social and economic interests [8], [10], [12]. A population target is ultimately determined by the ‘actors’ involved in the decision-making process by outlining their interests and then negotiating an acceptable outcome that mutually satisfies all their interests. Such decision-making is sensitive to potential imbalances of power between stakeholder groups [24]. For instance can the interests of all those involved in the decision-making process be accommodated, and do the interests of certain ‘actors’ have greater influence or override those of others? In addition, do the interests of the ‘actors’ fully represent the interests of stakeholders not involved in the decision-making? [14], [15]. As a number of interviewees made reference to in this study the likely reactions of the general public should also be considered. Public opinion can have considerable bearing on what is perceived, amongst both actors and non-actors, as an acceptable population target to control a waterbird species, as well as what are desirable and proportionate management actions to achieve it [25].

Clearly a challenge for those leading an adaptive management process is gaining general stakeholder acceptance where there are diverse groups with different interest. It becomes more problematic when endeavouring to involve stakeholders across wide spatial scales and at different institutional levels e.g. local, national and international [8]. This is particularly relevant when considering population control of trans-boundary migratory waterbird species. A key lesson from this study is the need for multi-level communication of information and data that underpins management decisions, as well as continuous monitoring of indicators to determine the impact of management actions. Science has an important role to play in providing relevant and useful information that outlines alternatives, clarifies choices and enables decision-makers to evaluate management actions to achieve desired outcomes [26]. Furthermore, other studies have emphasised that the process of providing and communicating scientific information, as well as defining and setting targets amongst stakeholders is just as important as to what the final targets are [7], [10], [26]. A flexible approach for stakeholder participation may be needed, using a variety of participatory methods, to encourage different stakeholder groups to learn from each other over time [14], [19]. In this way relevant stakeholder voices can come through at different stages of the adaptive management cycle enabling shared understandings to develop. This ‘social learning’ can facilitate the flow of information and knowledge between stakeholders both horizontally (among groups) and vertically (among institutional levels) [14]. These points highlight some of the challenges that face those responsible for developing management policies for migratory waterbirds to engage and communicate with diverse stakeholders at international and national levels.

The results of this study are drawn from interviewee perspectives in response to particular situations, predominately concerning the management of goose related conflicts. It is important to understand the context of a situation to be managed and how it is viewed by those involved. For instance, the Svalbard pink-footed goose International Species Management Plan (ISMP) is seen as a potential model for waterbird management within Europe. A population target has been set based on a preliminary demographic population modelling [27] but also including stakeholder views on acceptable minimum and maximum population sizes. International cooperation has been essential to the successful progress of the Svalbard Pink-footed Goose ISMP so far, facilitated by the involvement and collaboration of a variety of international and national stakeholders [6]. Such ISMPs are intended to offer a structured, integrated and collaborative management approach working towards unified goals and objectives. However, it is recognised that the population target may need to be revisited in coming years depending on what is learnt about population dynamics, ecological changes (e.g. artic degradation), economic impacts (e.g. crop damage) and the effects of management actions (e.g. hunting). Monitoring data and scientific knowledge can inform and improve stakeholder understanding when learning is shared between groups. The implication for those involved in adaptive management is to maintain a flexible view not only of stakeholder participation but also the setting of management objectives. Only in this way can management objectives evolve and adapt to changing ecological, social and economic imperatives over time.

This study indicates that an important part of the processes when setting population targets is the engagement of stakeholders: to align interests and to facilitate a shared understanding amongst those involved about the ecological, economic and management issues they collectively face. The challenge for government agencies is how best to engage with diverse groups of stakeholder. There is a growing recognition amongst scientists and wildlife managers to adopt a multi-disciplinary approach to bridge the science-society boundary, as well as utilise participatory approaches that engender conditions for social learning and enable stakeholders to work towards the co-management of problematic situations [14], [19], [28], [29].

## Conclusions

The setting of population targets for waterbird control is problematic because varying perspectives, particularly strongly held views about the need for population control, have implications when wishing to align the interests between diverse stakeholders, who may have different aims and objectives for managing a waterbird species. Nevertheless, the setting of a population target can be conceived as a unifying measurable objective but it must be recognised as just one possible measure for monitoring and evaluating management actions. There may well be multiple supporting objectives that encapsulate the management aims of different stakeholders. A population target and any supplementary measurable objectives should be linked to management issues and actions to aid the acquisition of knowledge, helping to determine progress towards an overall goal. The implication when considering the wide-scale control of waterbird species, is that in cases where it is likely that the population size matters any population target should be coupled to the issues being addressed, e.g. the scale of eco-system degradation or economic burden. Since setting a population target for controlling waterbirds is controversial, it should be accompanied by clear management statements about the situation, its context and the rationale for its use.

This study confirms that stakeholders wish to actively participate in the decision-making process but they generally expect government agencies to take the lead, developing policies and setting management objectives that reflect their goals and priorities that encompass biological, economic and social considerations. It is evident from this study that stakeholders regard participation and political discourse as important; not only to give consent but also to share knowledge and learning, which helps all involved understand the rationale for contentious actions as well as providing opportunities to influence management decisions. When setting population targets a key requirement is the need for scientific data and the continuous monitoring of management actions and their impacts in order for effective evaluation. This is expected and demanded by stakeholders. As has been suggested a flexible conceptualisation of participation is required to facilitate social learning, whereby the flow of information and knowledge can be transferred between groups, at different institutional levels as well as at different stages of an adaptive management cycle. However, it is uncertain from this study how this might be achieved in reality. We conclude that further work is needed to identify, develop and implement the institutional capacity and structures that can employ interdisciplinary and participatory approaches for engaging stakeholders at various levels, when making decisions and setting population targets for the management and control of waterbird populations.
